# Loose Prescribing

**Published:** 1903-02-28

**Authors:** 


### The Hospital.

A Journal of

### The Medical Sciences and Hospital Administration.

Vol. XXXIII.—No. 857. | Edited by Sir Henry Burdett, K.C.B., and Solomon C. Smith, M.D., M.R.C.P. | [February 28, 1903.

### Loose Prescribing.

Perhaps it is the spread of education, leading doctor and patient to talk over "the case" almost as if they were brother chips in consultation; perhaps it is the overwhelming multitude of ready-compounded drugs—tabloids, syrups, elixirs, and other more or less pleasant and easy modes of taking physic; perhaps it is the extension of the "stores" system, which makes patients try to shirk the expense of a properly-dispensed bottle of medicine; but whatever may be the cause, there can be no doubt that the old style of prescription, with all its niceties and particularities, is going somewhat out of fashion. The up-to-date patient regards it rather as a slur upon his intelligence if the whole pathology and treatment of his case is not explained to him, and the doctor is, perhaps, to be forgiven if, after such a frank exposition of his aims, he hesitates to indulge in any such "hocus pocus" as a prescription. He therefore, instead of writing out his formula in full, tells his patient to take a little of this, that, or the other, and in so doing he runs many risks. Not only does this easy-going plan involve danger to his patient, but it may prove most injurious to his own reputation. A patient who recovers under the use of a bismuth tabloid, which he can buy by the gross at any stores, is pretty sure to regret that he spent so heavy a fee for so trivial a malady; while, should he fail to recover, he will not hesitate to say that the doctor must have entirely failed to understand his case, or else have been guilty of culpable carelessness in treating it so lightly! Again, it is easy to see how the loose prescription of some ready-made medicine encourages patients in the bad habit of doing a little mutual quackery among themselves. Any man can understand that there is something personal and special in a formal prescription written for himself. Each ingredient has evidently been considered in reference to his own case. But the matter is quite different when a proprietary medicine or ready-made compound is ordered. With it the patient feels that he has got not so much a prescription for himself as a recipe for his ailment, and that so far as that ailment is concerned he is quite capable of showing all his friends how to treat themselves. This is the beginning of much trouble.

Chloral is doubtless a very useful drug, and it keeps very well in the form of syrup; but it is a frightfully dangerous thing to order syrup of chloral to be measured out by the patient, or even the patient's wife, as it may be required. A dose of morphia properly dispensed, and taken perhaps without the patient knowing even what it is, may give relief when nothing else avails; but how many lives have been ruined and lost in consequence of patients knowing the nature of the drug and learning the fatal art of self-medication by means of the hypodermic syringe! Every lady who has a house nowadays has also a medicine chest, and if, when called to an emergency, the doctor would shut himself up with it and compound out of its resources a medicine which will give relief, a medicine chest would be a very useful "household requisite." But when the doctor tells its owner to give, maybe to her child, a calomel powder, or to her daughter a little antipyrin, or to her husband a few drops of colchicum, the foundation is laid of a dangerous system of domestic drugging which is sure to lead to harm if not to actual disaster. In fact the evil consequences which spring from this unfortunate habit of giving verbal directions, and telling patients to procure ready-compounded medicines, instead of writing and signing a formal prescription to be properly made up and sent out with full written directions, are too obvious to need demonstration.

No doubt the charges made by some chemists for the compounding of prescriptions has had something to do with the development of this loose way of ordering drugs. If a patient's means are small it seems almost a shame to order a bottle of medicine which will have to be repeated every few days when, as may happen sometimes, a teaspoonful of bromide in a little water taken every day will do pretty nearly what is required. Then, unless one has great confidence in the dispenser, one cannot be sure that the extempore compound will be quite as nice or as easily taken as the patent, soluble, tasteless, compressed, sugar-coated affair that is prepared by the wholesale manufacturer. So well is this known that patients constantly ask, "Cannot I have it made up as a tabloid?" The same thing applies to many proprietary syrups, which are not only better to take, but "keep" far better than similar medicines made up according to extempore prescriptions. Lastly there is the question of uniformity. A medical man who orders some so-called patent preparation often does so, not because he thinks that it is better than anything for which he could write a formula, but rather because by so doing he ensures that wherever the patient may be he can obtain practically the same article, which is by no means always the case when a prescription is made up by different chemists. Hence the doctors have much excuse; but it is an evil habit, and often dangerous withal.

